# Bond strength of conventional versus modified methods for Class IV restorations in primary incisors: An in vitro study

**DOI:** 10.34172/joddd.2022.021

**Published:** 2022-10-15

**Authors:** Naser Aslaminabadi, Zohreh Halimi Tabrizi, Ozra Golsanamlou, Zohreh Estaki, Zahra Jamali

**Affiliations:** ^1^Department of Pediatric Dentistry, Faculty of Dentistry, Tabriz University of Medical Sciences, Tabriz, Iran; ^2^Department of Pediatric Dentistry, Faculty of Dentistry, Qazvin University of Medical Sciences, Qazvin, Iran; ^3^Department of Oral Medicine, Faculty of Dentistry, Tabriz University of Medical Sciences, Tabriz, Iran

**Keywords:** Composite resin restoration, Early childhood caries, Shear bond strength, Tensile bond strength

## Abstract

**Background.** Aesthetic restorations of severely decayed anterior primary teeth are challenging due to the small size of the teeth and the large pulp chambers. Therefore, this study evaluated and compared the tensile and shear bond strengths of conventional versus modified methods (slot technique) of Cl IV restorations in primary incisors.

**Methods.** A total of 120 extracted intact maxillary primary central and lateral incisors were divided into two groups. In group A, conventional Cl IV cavities were prepared. In group B, after conventional cavity preparation, four and three incisal slots were prepared on the incisal edges of the central and lateral incisors, respectively. All the teeth were restored using composite resin, and then the tensile and shear bond strengths were evaluated.

**Results.** A statistically significant increase in the tensile bond strength of restorations was recorded in the modified technique compared to the conventional method (*P*=0.001). Although an increase in the shear bond strengths was found in the modified method, the value did not reach a statistically significant level (*P*=0.158). The most frequent fracture type was adhesive in the conventional group and cohesive in the modified group, considering the tensile and shear bond strength tests. In both groups, the highest average tensile bond strength was recorded in teeth with the cohesive fracture in dentin, and the lowest average was seen in the adhesive type.

**Conclusions.** Incisal slots could increase the tensile bond strengths of Cl IV composite resin restorations in primary teeth.

## Introduction

 Early childhood caries (ECC) is a severe and rapidly developing type of dental caries that first begins in the cervical third of the maxillary incisors and subsequently causes complete destruction of the crown.^1^ Anterior tooth decay at an early age can lead to aesthetic problems, parafunctional habits, speech and psychological problems, decreased chewing efficiency, and decreased vertical occlusal dimension. Therefore, maintaining the integrity of deciduous teeth until they naturally exfoliate is essential for the proper development and maturity of the child, the growth of the facial skeletal complex to its ultimate potential, and the re-establishment of normal occlusion with the desired aesthetics.^2,3^

 However, cosmetic restorations in severely decayed anterior deciduous teeth are challenging due to the small size, the large pulp chambers, behavioral control problems in children, and the lack of sufficient enamel and dentin thickness, leading to a consequent inadequate bonding. Moreover, gingival inflammation due to poor oral hygiene and excessive bleeding after caries removal in patients with ECC makes cosmetic composite resin restoration in deciduous teeth more challenging.^4,5^Furthermore, conservative cavity preparation in deciduous teeth leads to a lower thickness of the restorative materials, consequently increasing the restoration failure due to lower resistance.^6^ On the other hand, it has been shown that the caries process has a detrimental effect on the mechanical properties of dentin in anterior deciduous teeth, which further increases the likelihood of restorative failure.^7^ Besides, given the behavioral problems of young children during dental treatment, it is necessary to minimize the duration of the dental session.

 Various studies have introduced several methods for the Cl IV restorations of permanent anterior teeth, and over time, different preparation techniques have been developed depending on the materials used. These techniques include the preparation of butt joint margins,^8^ 45-degree bevels, slot preparation,^9^ chamber preparation,^10^ short bevel preparation,^11^ and long bevel preparation to improve aesthetics.^12^ The available studies support using adhesive bonding materials to increase the retention of the restoration and reduce leakage and sensitivity of deciduous and permanent teeth. The clinical success of adhesive restorations has led to more conservative preparation of teeth in the case of composite resin restorations.^13^ However, the lack of sufficient dentin structure due to the large pulp chamber, and the thin layer of prismless enamel that makes etching difficult, make the adhesive systems less effective in primary teeth.^14,15^

 Mechanical locks or slots are recommended in primary teeth to overcome the above-mentioned bonding challenges. These locks and slots are prepared on the labial or lingual surfaces and improve the retention of the restoration by increasing the bonding area.^16^ However, further studies are necessary to evaluate these methods.^13^

 Given the limitations of the conventional adhesive techniques in restoring anterior deciduous teeth and the lack of a comprehensive strategy to render a proper restoration in decayed deciduous teeth, this study aimed to introduce and test a new Cl IV preparation method, with additional slots on the incisal edge.

## Methods

###  Specimen preparation

 We used 120 intact deciduous central and lateral incisor teeth extracted due to orthodontic reasons such as lingual eruption or over-retention. They were immediately kept in 0.2% thymol forfourdays for disinfection and were then stored in normal saline solution at room temperature until tested. Soft tissue residues and plaques were carefully removed from tooth surfaces using rubber cups and a water-pumice slurry with a low-speed handpiece. Then, the teeth were mounted 2 mm below the CEJ (approximately at the level of the alveolar bone in an intact tooth) in self-cured acrylic resin in cylindrical plastic molds while adjusting the labial tooth surface parallel to the walls of the plastic mold.

 The cavities were standardized according to the established protocols, including an incisogingival dimension of 4 mm, a cavity depth of 1 mm, a mesiodistal dimension of 5 mm (in central incisors) and 4 mm (in lateral incisors), and a buccolingual dimension of 3 mm.^17^ An attempt was made to allow the same thickness of cavity wall and tooth structure in each group using an orthometer gauge (Korkhaus Orthometer Kit, 75228 Ispringen, Dentaurm, Germany). A class IV cavity was prepared in each tooth using a high-speed handpiece (NSK, Tokyo, Japan) and a diamond fissure bur (No. 138, D&Z, Wiesbaden, Germany) under water spray. The diamond burs were replaced after five cavity preparation procedures.^18^

**Figure 1:**
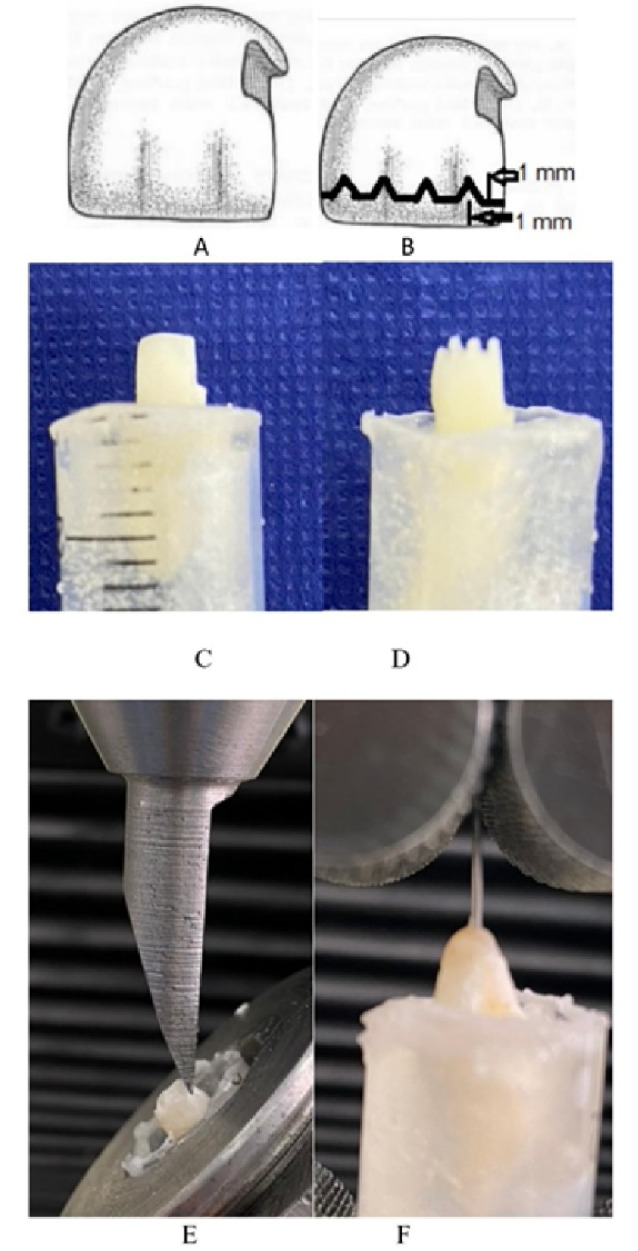
Preparation methods. A: Schematic representation of conventional preparation. B: Schematic representation of modified preparation. C. Conventional preparation. D: Modified preparation. E: Sample in the universal testing machine for measuring shear bond strength. F: Sample in the universal testing machine for measuring tensile bond strength.

 The samples were randomly categorized into two groups (Table 1). In group A, the preparation of Cl IV cavities was performed with a 2-mm wide cavosurface margin at a 45° angle to increase the level of the etched enamel (Figure 1A). In group B, after conventional cavity preparation, three and four incisal slots were prepared on the incisal edges of the lateral and central incisors, respectively. The depth of these slots was 1 mm (1 mm below the incisal edge), the length of the slots was equal to the diameter of the bur, and the distance between the slots was 1 mm (Figure 1B). These slots were created with the tip of a #858 needle-shaped dental bur with a head length of 8 mm, a head diameter of 1.4 mm, and a tip diameter of 0.6 mm. Then the sharp edges of the slots were rounded.

**Table 1:** Distribution of selected teeth

**Preparation type**	**Central**	**Lateral**	**Total**
A	32	28	60
B	32	28	60
Total	64	56	120

 After completing the cavity preparation procedures, all the teeth were etched with 37% phosphoric acid gel (Morva Etch, Iran) for 30 seconds. The cavities were rinsed with air and water spray for 20 seconds and gently air-dried to keep the tooth surface moist. The two-stage adhesive system (Denfil BC Plus, South Korea) was applied in two successive layers with a clean microbrush (Microbrush Premium Plus, China) and dried gently for 5 seconds. Then, it was polymerized with a light-curing device (Bluephase® C5, Ivoclar Vivadent, Schaan, Liechtenstein) with an output of 2400 mW/cm^2^ for 20 seconds.^17,19^

 The anterior composite resin (Charisma Topaz, Kulzer, Germany) was then placed on the preparation and cured for 40 seconds.^14^ A transparent matrix piece was placed on the last layer to remove excess material and speed up the polishing process. Finally, the samples were polished using a diamond polishing bur (D&Z, Wiesbaden, Germany) and polishing discs (EVE Flexi-D, Germany) with water spray. To simulate the aging condition of the oral cavity, the samples were placed in water baths in a thermocycler for 500 cycles between 6°C and 60°C with a dwell time of 30 s per bath.^20-22^

 The samples were stored in distilled water at room temperature until all the specimens were prepared for the tensile and shear bond strength tests. Then, all the samples in groups A and B were randomly assigned to subgroups 1 (A1 and B1) and 2 (A2 and B2) for the tensile and shear bond strength tests_,_ respectively.

###  Tensile and shear bond strengths

 To assess the tensile bond strength, the teeth were fixed in the metal base of the Instron device, and the force lever of the machine applied the force to an omega-shaped wire (Ω), which was designed and made with an 0.5-mm stainless steel wire. The omega-shaped part was above the incisal edge of the tooth, and the long-hooked arm of the omega-shaped wire was embedded in the composite resin, parallel to the tooth longitudinal axis. Then, the force was increasingly applied along the tooth longitudinal axis at a strain rate of 12.7 mm/min and continued until the restoration detached. This force value was recorded as the tensile bond strength (Figure 1F).

**Figure 2:**
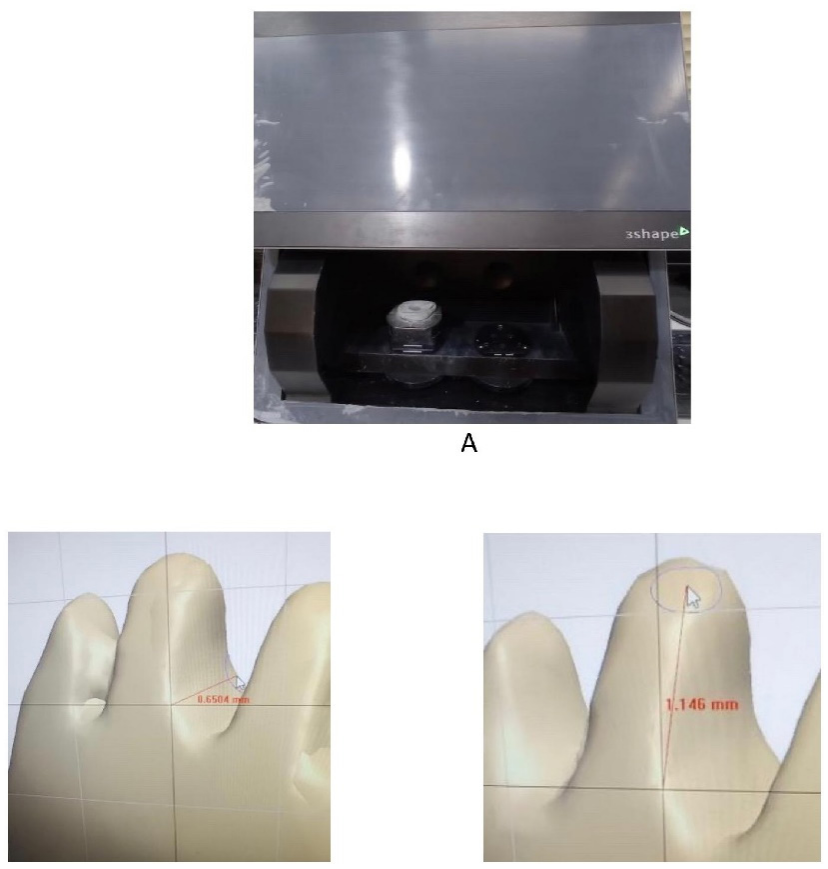
(A) The 3Shape scanner. (B & C) Lines are drawn to measure the bonded surfaces.

 The shear bond strength of the restoration was evaluated by applying an inclined force at an angle of 45° to the longitudinal axis of the tooth in a universal testing machine (Instron). The compressive force was increasingly applied to the proximal surface at a crosshead speed of 12.7 mm/min and continued until the restoration detached. This value was measured as tension output,^23^ (Figure 1E). The shear bond strength was calculated from the formula S = T/A, in which S is the shear bond strength, T is the tensile input, and A is the bonded area.^24^ The bonded surface was calculated by 3Shape software. First, the desired tooth was selected in the order section of the software; then, the prepared tooth was scanned in Trios scanner (3Shape, Copenhagen, Denmark). Using the measurement grid option, the desired surface was marked by drawing lines, and the surface area was calculated by the software (Figure 2). Finally, the samples were examined to determine the mode of failure, which was categorized as adhesive (at the interface between teeth and composite resin), dentin cohesive (inside dentin), and composite cohesive (inside the composite resin material).

###  Statistical analysis

 The normality of the data was evaluated by the Kolmogorov-Smirnov test. Parametric one-way ANOVA and independent *t*-test were used to compare the preparation methods and types of fractures. Two-way ANOVA was used to investigate the simultaneous effects of fracture type and preparation method on the shear and tensile bond strength. Finally, post hoc Tukey tests were used to determine the differences between the two groups.

## Results

 A total of 120 primary incisors were included in this study (32 central incisors and 28 lateral incisors in each group).

###  Relationship between the preparation method and bond strengths

 A statistically significant increase was recorded in the tensile bond strength of restorations in the modified group compared to the conventional method (*P* = 0.001). Although an increase was found in the shear bond strengths of the modified method, the value did not reach a statistically significant level (*P* = 0.158) (Table 2).

**Table 2:** Comparison of shear bond strength and tensile bond strength between preparation methods

**Preparation method **	**Number **	**Shear bond strength **	***P*** ** value **	**Tensile bond strength **	***P*** ** value **
Conventional	30	207.8 ± 63.22	0.158**	34.61 ± 14.23	0.001*
Modified	30	231.86 ± 66.88		48.24 ± 14.43	

*The statistical analysis (one-way ANOVA and independent t-test, *P* < 0.05) of the tensile bond strength for the two groups indicated a significant increase in the modified group. **However, no significant difference was found in the quantity of the mean shear bond strength between the conventional and the modified groups.

###  Relationship between force magnitude and fracture type

**Figure 3:**
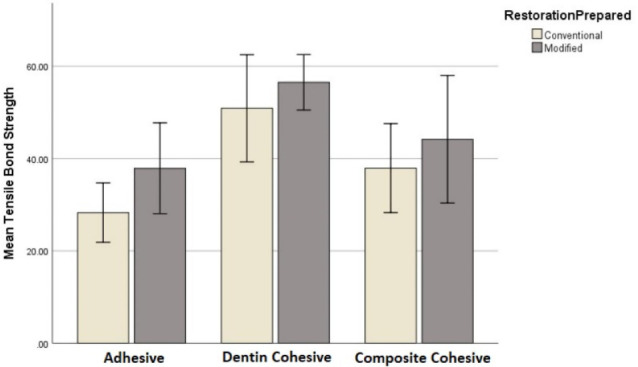
Comparison of the tensile bond strength between the methods and the fracture type.

**Figure 4:**
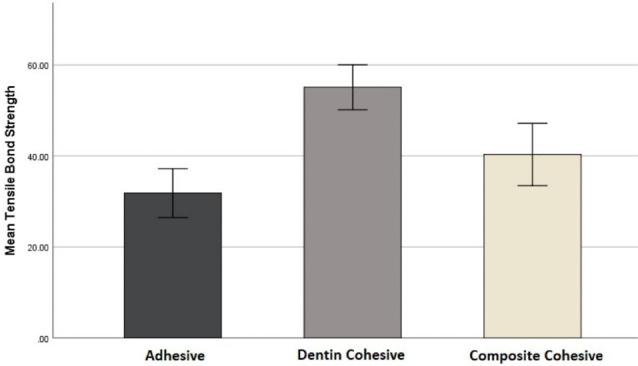
Comparison of the shear bond strength between the methods and the fracture type.

Table 3 shows the most frequent type of fracture in each subgroup. In the tensile bond strength evaluation, the most frequent type of fracture in conventional (A1) and modified technique (B1) subgroups were adhesive and dentin cohesive, respectively. In both subgroups (A1 and B1), the highest mean tensile bond strength value was recorded in cohesive dentin fracture, and the lowest mean was recorded in the adhesive type (Table 3; Figures 3 and 4).

**Table 3:** Comparison of tensile bond strength between types of fractures and preparation methods

**Preparation method**	**Fracture type **	**Number **	**Tensile bond strength**
Conventional	Adhesive	17	28.27 ± 12.5
	Dentin cohesive	5	50.88 ± 9.36
	Composite cohesive	8	37.92 ± 11.52
	total	30	34.61 ± 14.23
Modified	Adhesive	10	37.89 ± 13.77
	Dentin cohesive	15	56.51 ± 10.87
	Composite cohesive	5	44.16 ± 11.13
	total	30	48.24 ± 14.43

**Figure 5:**
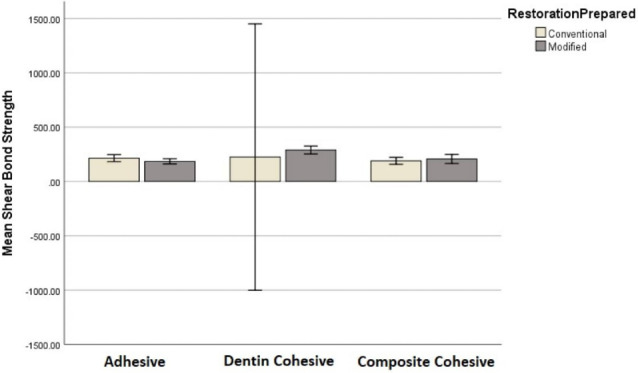
Comparison of the tensile bond strength between the methods and the fracture type.

 Considering shear bond strength, the most frequent type of fracture was adhesive in the conventional group (A2) and dentin cohesive in the modified group (B2). In addition, the highest mean value of shear bond strength in both groups (A2 and B2) was seen in cohesive dentin fracture. However, the lowest mean for the conventional group (A2) was recorded in the adhesive type. This value for the modified group (B2) was seen in cohesive composite fracture (Table 4, Figure 5).

**Table 4:** Comparison of shear bond strength between types of fractures and preparation methods

**Preparation method **	**Fracture type **	**Number**	**Shear bond strength**
Conventional	Adhesive	19	214.67 ± 66.37
	Dentin cohesive	2	224.3 ± 136.47
	Composite cohesive	9	189.61 ± 41.21
	Total	30	207.8 ± 63.22
Modified	Adhesive	11	185.03 ± 35.41
	Dentin cohesive	12	289.44 ± 57.77
	Composite cohesive	7	206.72 ± 44.98
	Total	30	231.85 ± 48.43

###  Relationship between different types of fractures in terms of the tensile and shear bond strength

 Considering tensile bond strength, one-way ANOVA was used for the pairwise comparison of the mean magnitude of the three different fracture types in each group (A1 and B1) separately. A significant difference in both conventional and modified groups was recorded. Post hoc Tukey tests were conducted to determine the differences between the two specific fractures. The difference was significant only between dentin cohesive with adhesive (*P* < 0.001) and dentin cohesive and composite resin cohesive fractures (*P* = 0.023). This value was not statistically significant between composite resin cohesive and adhesive fractures (*P* = 0.179) (Table 5).

**Table 5:** Comparison of tensile bond strength between types of fractures

**Fracture type **	** Difference**	***P*** ** value**
Adhesive- dentin cohesive	-20.61	< 0.001*
Adhesive- composite cohesive	-7.95	0.179**
Dentin cohesive- composite cohesive	12.65	*0.023

*Tukey’s post hoc test (*P* < 0.05) was conducted to determine the differences between each of the two specific fractures, which demonstrated that this difference was significant only between dentin cohesive and adhesive and composite cohesive, **but not significant between composite cohesive with any of the other fractures.

 Considering shear bond strength, one-way ANOVA demonstrated a statistically significant difference in the modified group (A2) but not in the conventional group (B2) when the mean shear bond strengths of the three fracture types were compared in each group separately. The Games-Howell post hoc tests were conducted to compare all the possible combinations of differences between the groups. The results suggested that the mentioned difference was significant in the dentin cohesive with composite resin cohesive comparison and the dentin cohesive with adhesive comparison (*P* < 0.001 and *P =*0.009). This value was not significant in the composite resin cohesive–adhesive comparison (*P* = 0.545).

###  Cumulative effect of the fracture type and the preparation method on the shear and the tensile bond strengths

 A two-way ANOVA was conducted to investigate the cumulative effect of the fracture type and preparation method on the shear and the tensile bond strengths. Considering the shear bond strength, the results indicated that the two variables did not have a statistically significant effect either separately (*P* = 0.054, *P* = 0.352) or simultaneously (*P* = 0.114). Considering the tensile bond strength, although no statistically significant cumulative effect was seen by the mentioned variables (*P* = 0.851), the separate effect of the variables seemed to be statistically significant (*P* < 0.001, *P* = 0.042).

###  Relationship between the tooth type and the fracture type

 There was a significant relationship between the tooth type and the fracture type, considering the shear bond strength values (*P* < 0.001). The most common fracture type was adhesive in the central incisors, with composite resin cohesive in the lateral incisors.

 When considering the tensile bond strength test, the most common fracture type was the adhesive fracture in both central and lateral incisor teeth; however, the relationship between the tooth type and the fracture type was not statistically significant (*P* = 0.977).

## Discussion

 The present study aimed to introduce a new preparation method for Cl IV cavities in anterior primary teeth. This in vitro study evaluated and compared the tensile and shear bond strength of conventional versus modified method (slot technique) of Cl IV restorations in primary incisors. The tensile and the shear bond strengths were evaluated in 120 lateral and central incisor teeth.

 Considering the tensile and shear bond strengths in different preparation methods, although the shear bond values did not reach a statistically significant level, higher tensile and shear bond strength values were observed in the modified method (slot technique). The higher bond strength values in the modified technique can be attributed to the increased enamel and dentin surface areas available for bonding; therefore, a greater force is required for debonding.^25^ Suzuki and Finger^26^ reported that thearea of sound dentin available for bonding was one of the main factors influencing the tensile bond strength. The slot technique might also increase the mechanical retention of composite resin restorations, leading to higher tensile bond strength.^27^

 The most frequent fracture type was adhesive in the conventional group and dentin cohesive in the modified group. In both groups, the highest mean tensile bond strength was recorded in the cohesive dentin fracture, and the lowest magnitude was seen in the adhesive type. Regarding shear bond strength in the conventional group, the highest mean was recorded in dentin cohesive type, with the lowest in composite cohesive type. These values for the modified group were dentin cohesive type and adhesive type, respectively.

 According to the results, the highest amount of force was recorded in dentin cohesive fracture in both preparation methods and strength tests. The results showed that the most common type of fracture in higher forces was dentin cohesive regardless of the preparation method. This might be because when the bond between the tooth surface and composite resin is weak, the debonding occurs at lower forces, and adhesive fracture is seen. On the other hand, more force is needed to overcome this bond when there is a stronger bond between the tooth surface and composite resin. At one point, the magnitude of the applied force approaches a level higher than the flexural strength of dentin, and cohesive dentin fracture occurs. These findings are consistent with previous studies, which indicated that more force is required for cohesive dentin fracture than other fracture types, and adhesive failures occur in lower bond strength values.^28^ However, some studies did not find a significant relationship between the fracture type and the magnitude of the bond strength.^29-31^

 In a study by Pithan et al^32^ on the tensile bond strength of intracanal posts in deciduous anterior teeth, the most frequent type of fracture was adhesive. Their study suggested that the most important factor is the bond between the adhesive systems and root canal walls, not the type of intracanal retention used.

 Considering the shear bond strength magnitude in all the samples, this value was independent of the preparation method and fracture type, either alone or simultaneously. Considering the tensile bond strength magnitude, this value was independent of the cumulative effect of preparation method and fracture type; however, it depended on the preparation method and fracture type separately. The main reason for this is that shear bond strength is calculated based on the bonded area; therefore, the difference in the bonded area between the two preparation methods does not affect the shear bond strength. According to previous studies, the tensile bond strength is directly influenced by the degree of adaptation of the composite resin on the prepared tooth surface.^32^ Using the modified preparation method and creating retentive slots could increase the possibility of placing composite resin in the incremental layer, leading to the close adaptation of composite resin to the prepared tooth.

 There was a statistically significant relationship between the tooth type and the fracture type in shear bond strength values. The greater frequency of cohesive composite resin fracture in the lateral teeth might be due to the lower preparation surface and lower volume of composite resin used for restoration in the lateral incisor compared to the central incisor, leading to lower resistance of restoration against the shear force.

 All the samples in this study were prepared with a standard Cl IV preparation to determine the effect of slot preparation on bond strength. However, slot preparation could also be used in other types of cavities, including severely damaged anterior teeth, with several advantages compared to previously introduced restorative methods. For example, the intracanal pin technique could interfere with root resorption, resulting in cracks and strains in the root structure.^32^ These issues will be overcome when the adhesive restorative methods like the slot technique are used. Another important issue is the duration of the treatment session, which is very critical due to the behavioral considerations of children. Using intracanal pins and creating resistant retention slots in the root are time-consuming, and the desired results might not be achieved due to the disruption of the child’s cooperation.^32,33^ However, adhesive restorative techniques have less chair-side time and can be performed for several teeth in a short time.

 Another problem is the additional materials and tools required for intracanal pins or intracanal slots, such as special burs and pins, which might impose additional costs. However, the slot technique could be done with commonly used burs and easily integrated with intracanal composite resin and fiber-based posts. Further studies are necessary to assess the combined effect of the slot technique with intracanal posts.

## Conclusion

 It can be concluded from the results that implementing the slot technique (modified method) in Cl IV composite restorations might increase the tensile bond strength of the restoration and decrease the risk of the adhesive and composite resin cohesive fractures. However, the mentioned technique did not exhibit any significant difference in the shear bond strength of the restorations.

## Acknowledgments

 The authors would like to thank the members of the Pediatric Dentistry Department, Faculty of Dentistry, Tabriz University of Medical Sciences, for their help and support in the implementation of this study.

## Authors’ Contribution

 NAA and ZH contributed to the study’s design and conception. ZH collected data. All authors were involved in the drafting and final approval of the manuscript.

## Funding

 This study was supported and funded by Tabriz University of Medical Sciences.

## Ethics Approval

 Not applicable.

## Competing Interests

 The authors declare no competing interests and no conflict of interests.
